# Multiple Inflammatory Scalp Nodules in an Infant: A Diagnostic Challenge

**DOI:** 10.1002/ccr3.73243

**Published:** 2026-07-28

**Authors:** Chukwuka Elendu, Mbanefo C. Uyanwune

**Affiliations:** ^1^ Federal University Teaching Hospital Owerri Nigeria; ^2^ City St George's, University of London London UK

**Keywords:** diagnostic challenge, follicular disorders, infant, inflammatory scalp nodules, pediatric dermatology

## Abstract

Multiple scalp nodules in infancy warrant cautious interpretation, as their extent and pattern may signal conditions requiring broader evaluation than localized infection. Avoiding premature diagnostic labels and relying on careful visual assessment helps ensure timely investigation, appropriate therapy, and prevention of long‐term cutaneous complications.

## 1 Case Description

**FIGURE 1:**
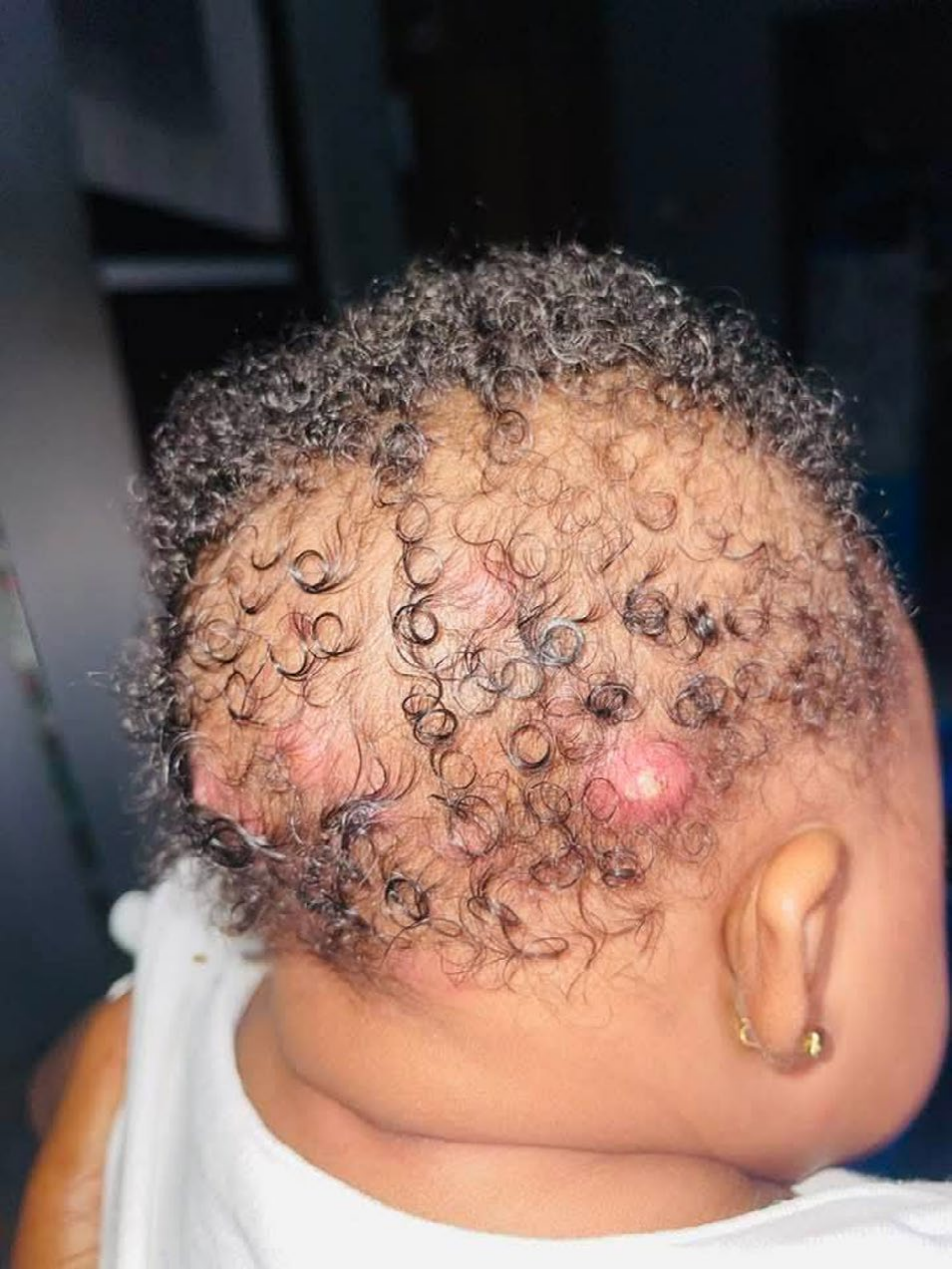
Multiple erythematous inflammatory nodules and pustules involving the occipital scalp of an infant, with background scalp erythema, perifollicular inflammation, and patchy hair thinning.

A 9‐month‐old female infant was evaluated because of progressive scalp lesions noted over 5 days. Physical examination revealed multiple discrete and confluent inflammatory nodules and pustules distributed over the occipital and parietal scalp. The lesions were dome‐shaped, erythematous, and tender, with some showing central pustulation. Surrounding areas demonstrated diffuse scalp erythema, perifollicular inflammation, and patchy hair thinning, suggesting follicular involvement (Figure 1). No ulceration or overt necrosis was observed, and there was no obvious draining sinus at the time of assessment.

The infant was febrile, with a temperature of 38.4°C, and irritable at presentation, but appeared clinically stable with no features of systemic toxicity. There was no visible cervical or occipital lymphadenopathy and no evidence of trauma to the scalp. The remainder of the cutaneous examination was unremarkable, with no similar lesions identified on the face, trunk, or extremities. There was no history of prior topical or systemic treatment before presentation, and the scalp nodules varied in size and stage.

Given the diagnostic uncertainty and the presence of fever at presentation, bacterial swab cultures from the pustular lesions and potassium hydroxide (KOH) examination were performed, yielding growth of *Staphylococcus aureus* and no fungal elements, respectively. Basic laboratory evaluation, including complete blood count and C‐reactive protein, revealed mild leukocytosis (18.4 × 10^9^/L [reference range: 6.0–17.5 × 10^9^/L]), neutrophilia (10.2 × 10^9^/L [reference range: 1.5–8.5 × 10^9^/L]), and elevated C‐reactive protein (32 mg/L [reference range: < 5 mg/L]), consistent with an acute inflammatory process. Based on these findings, a diagnosis of bacterial folliculitis/furunculosis was made, and the infant was commenced on oral flucloxacillin for 10 days, with marked improvement observed at follow‐up after 7 days, including reduction in inflammation and gradual resolution of the nodular lesions. At 4‐week follow‐up, near‐complete resolution of the lesions was observed, with no evidence of recurrence and progressive regrowth of hair in the previously affected areas.

## 2 Discussion

Multiple inflammatory scalp nodules in an infant represent a diagnostic challenge requiring a morphology‐driven approach; mislabeling such lesions as simple “boils” may obscure a broader inflammatory or infectious process and delay appropriate evaluation [1]. Key differentials include staphylococcal folliculitis/furunculosis, presenting with discrete tender nodules, central pustulation, and lesions at varying stages [2]; tinea capitis/kerion, characterized by tender nodules, alopecic patches, and marked inflammation warranting early fungal evaluation to prevent scarring, although this diagnosis was considered less likely because boggy plaques were absent and fungal studies were negative [3]; eosinophilic pustular folliculitis, characterized by recurrent sterile pustules and eosinophilic inflammation but considered unlikely because bacterial culture yielded *Staphylococcus aureus* and the lesions resolved with systemic antibiotics; pustular eczema with impetiginization, associated with an eczematous background and crusting, both absent; dissecting cellulitis–like presentations, characterized by deep‐seated nodules with sinus tract formation, absent on examination; scalp abscess related to trauma or occlusive practices, associated with localized fluctuant swelling and a history of trauma or occlusion, neither present; and, less commonly, Langerhans cell histiocytosis, presenting with persistent or treatment‐resistant lesions but considered less likely because of the rapid clinical improvement.

## Author Contributions

**Chukwuka Elendu:** conceptualization, data curation, formal analysis, investigation, methodology, project administration, supervision, validation, visualization, writing – original draft, writing – review and editing. **Mbanefo C. Uyanwune:** validation, visualization, writing – review and editing.

## Funding

The authors have nothing to report.

## Ethics Statement

The authors have nothing to report.

## Consent

Written informed consent was obtained from the patient's parent/legal guardian for publication of this report and the accompanying clinical image.

## Conflicts of Interest

The authors declare no conflicts of interest.

## Data Availability

All relevant clinical information is included within the article.
